# The DNTTIP1-PARP1 interaction orchestrates MiDAC recruitment and activity for NHEJ-mediated genome stability

**DOI:** 10.1016/j.jbc.2026.113342

**Published:** 2026-07-16

**Authors:** Yan Qin, Yanhui Ma, Ao Wei, Zhao Zhang, Peida Bao, Shaowei Jia, Mengyu Zhou, Ling Liu, Zhenzhen Yang, Lei Shi, Kaiwen Bao

**Affiliations:** 1 Key Laboratory of Breast Cancer Prevention and Therapy (Ministry of Education), Key Laboratory of Immune Microenvironment and Disease (Ministry of Education), The Province and Ministry Co-sponsored Collaborative Innovation Center for Medical Epigenetics, Department of Biochemistry and Molecular Biology, School of Basic Medical Sciences, Tianjin Medical University Cancer Institute and Hospital, Tianjin Medical University, Tianjin, China; 2 Tianjin’s Clinical Research Center for Cancer, Science and Innovation Center of Tianjin Medical University Cancer Institute and Hospital, National Clinical Research Center for Cancer, Tianjin Medical University Cancer Institute & Hospital, Tianjin, China

**Keywords:** CSR, DDR, MiDAC, NHEJ, PARP1

## Abstract

The Mitotic Deacetylase Complex (MiDAC) plays a crucial role in the DNA damage response (DDR), yet the mechanism of its recruitment to double-strand breaks (DSBs) remains poorly unclear. In this study, we identify Poly(ADP-ribose) polymerase 1 (PARP1) as the key factor that directs MiDAC to DSB-proximal chromatin. We demonstrate that this process depends on the physical presence of PARP1, but not its poly(ADP-ribosyl)ation (PARylation) activity. Specifically, the dimerization domain of the MiDAC subunit DNTTIP1 interacts directly with PARP1. Disrupting this interaction prevents MiDAC recruitment and impairs the deacetylation of histone H2A at lysine 5 and 9 (H2AK5ac/K9ac) at damage sites. This leads to genome instability, characterized by an increase in γH2AX foci, accumulation of DNA damage, and chromosomal breaks. The PARP1-DNTTIP1 interaction is mechanistically essential for the assembly of the non-homologous end joining (NHEJ) synaptic complex, as evidenced by a defective KU80-LIG4 interaction and impaired NHEJ efficiency when this interaction is disrupted. Consequently, this interaction is critical for physiological processes that rely on NHEJ, such as immunoglobulin class switch recombination (CSR) in B cells. Our findings establish the PARP1-DNTTIP1 axis as a critical PARylation-independent regulator of MiDAC, linking the sensing of early DNA damage to the subsequent remodeling of chromatin and the efficient repair of DSBs.

Genomic integrity is a fundamental cornerstone of cellular viability and function that is perpetually challenged by DNA-damaging agents, both endogenous and exogenous. Faithful lesion repair, particularly of toxic DSBs, requires the precise orchestration of damage sensors, signal transducers, and repair effectors (1, 2, 3). Key regulators of this chromatin landscape include histone deacetylase complexes, which tighten chromatin structure by removing acetyl groups. The MiDAC, comprising core subunits such as DNTTIP1, MIDEAS, HDAC1 and HDAC2, is one such complex that was originally associated with mitotic progression but is now also recognized as being involved in the DDR (4, 5, 6). Our previous work revealed that MiDAC localizes to chromatin flanking DSBs and facilitates DSB repair, with DNTTIP1 playing a central role (4, 7). However, the precise mechanism governing the recruitment of MiDAC to sites of DNA damage remains elusive.

Recent work by Bao *et al*. provided crucial functional insight, demonstrating that MiDAC catalyzes the deacetylation of H2AK5/K9ac. This specific modification appears to act as a licensing signal, rendering the local chromatin environment permissive for the stable assembly of the NHEJ synaptic complex (8). NHEJ is the dominant DSB repair pathway in mammalian cells, particularly during the G1 phase of the cell cycle. The process begins with the rapid binding of the KU70/KU80 (KU) heterodimer to the broken ends of the DNA. This then recruits the catalytic subunit of the DNA-dependent protein kinase (DNA-PKcs), forming the DNA-PK holoenzyme (9, 10, 11). This core then serves as a scaffold for the sequential recruitment of the ligation machinery (XRCC4, DNA ligase IV (LIG4) and XLF), forming a flexible 'long-range' complex that bridges the ends (12, 13). A critical transition, driven by DNA-PKcs autophosphorylation and partial dissociation, then compacts this structure into a 'short-range' synaptic complex that is capable of end alignment and ligation (14, 15). The finding that MiDAC-mediated deacetylation precedes and enables this complex assembly highlights MiDAC as a crucial upstream chromatin modifier for NHEJ; however, the trigger for its own recruitment remains unknown (8).

We therefore turned our attention to the earliest events in the DDR. PARP1 is a prototypical first responder to DNA strand breaks. It binds to DNA breaks, undergoes a dramatic increase in catalytic activity and synthesizes long, branched poly(ADP-ribose) (PAR) chains on itself and target proteins (16). This PARylation process creates a negatively charged scaffold that recruits a variety of repair factors *via* PAR-binding motifs (17). However, emerging evidence suggests that PARP1 also operates through non-catalytic scaffolding functions, facilitating protein–protein interactions independent of its enzymatic activity (18, 19). High-resolution microscopy, including super-resolution imaging and electron microscopy, has provided direct evidence that the Ku70/80 heterodimer and PARP1 are rapidly and independently recruited to DNA DSBs, acting as early damage sensors (20, 21). Similar to PARP1, the MiDAC complex also accumulates at DSBs with immediate kinetics. Given this parallel behavior and the fact that PARP1 can serve as a scaffold for multiple protein interactions, we hypothesized that PARP1 might be the upstream recruiter for the MiDAC complex. This would position PARP1 as a master regulator that initiates PAR-dependent signaling and directly mobilizes essential chromatin-remodeling activities.

In this study, we investigate the mechanism of MiDAC recruitment and its functional consequences. We found that PARP1, rather than its enzymatic activity, is essential for targeting MiDAC to DSBs *via* a direct interaction with the dimerization domain of DNTTIP1. Disrupting this interaction prevents not only MiDAC recruitment and associated histone deacetylation, but also compromises genome stability and the efficiency of the major DSB repair pathway: NHEJ. Furthermore, we demonstrate that this mechanism is vital for the physiology of immunoglobulin class switch recombination. Our study reveals a novel, activity-independent function of PARP1: recruiting a key chromatin modifier to DNA lesions to ensure accurate DSB repair and genomic homeostasis.

## Results

### MiDAC is oriented to DSB in a PARP1-dependent but PARylation-independent manner

**Figure 1:**
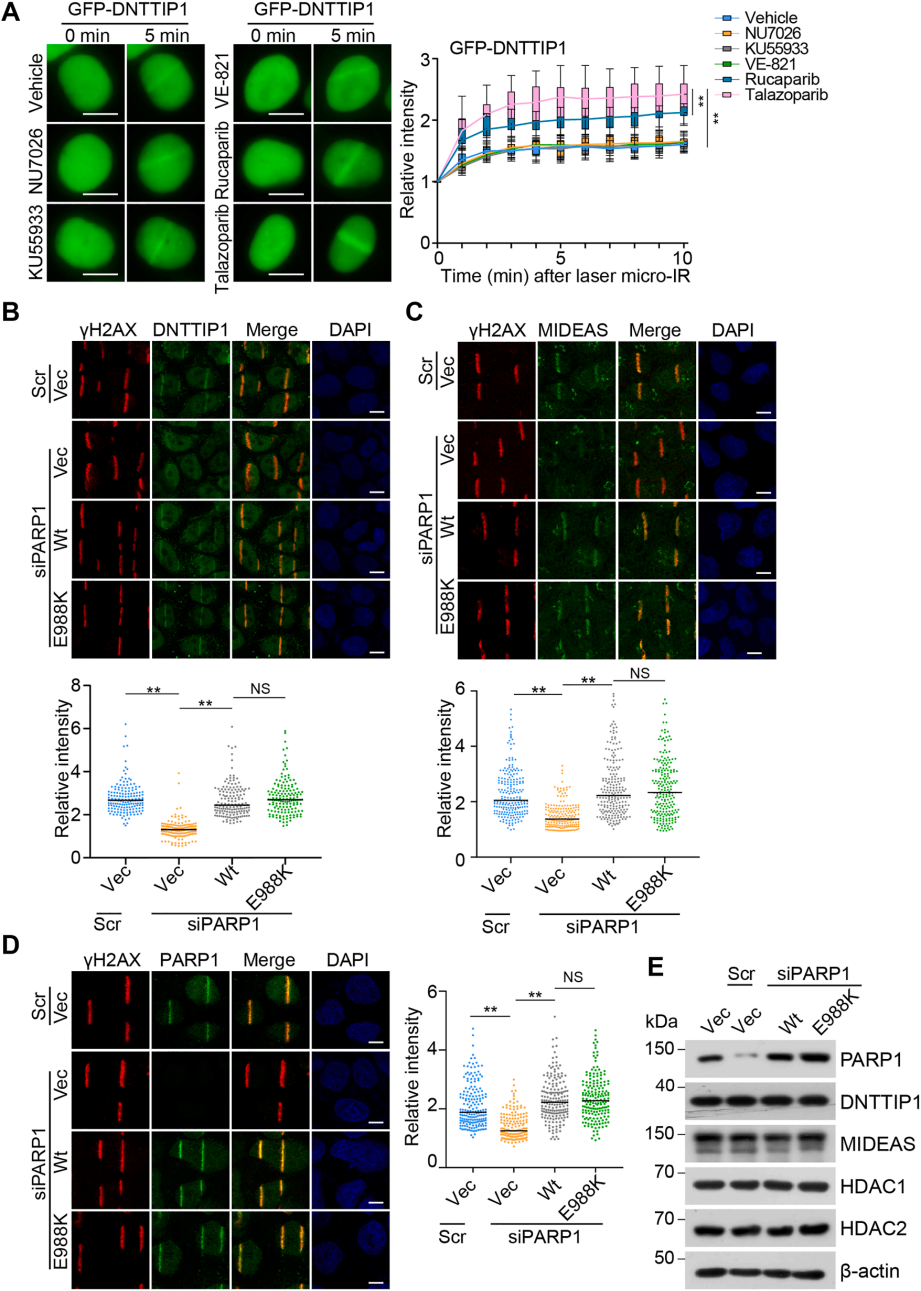
**MiDAC is oriented to DSB in a PARP1-dependent but PARylation-independent manner.***A*, recruitment kinetics of GFP-tagged DNTTIP1 were examined using laser micro-IR in HeLa cells that had been pre-treated for 4 hours with inhibitors against DNA-PKcs (NU7026, 10 μM), ATM (KU55933, 10 μM), ATR (VE-821, 10 μM) and PARP1 (Rucaparib and Talazoparib, both at 10 μM). The intensity of the DNTTIP1 signal at the laser stripes was quantified and normalized to that of the nuclear background (n > 15). Immunostaining was followed by confocal microscopy analysis of DNTTIP1 (*B*), MIDEAS (*C*), and PARP1 (*D*) recruitment at γH2AX-marked laser micro-IR regions in HeLa cells expressing PARP1 3′UTR siRNA and stably integrating PARP1/Wt or PARP1/E988K. The intensity of DNTTIP1, MIDEAS and PARP1 at γH2AX-marked DNA lesions was also quantified and normalized to the nuclear background (n > 100). *E*, PARP1 and MiDAC expression was analyzed by IB in HeLa cells expressing 3′UTR siRNA against PARP1 and the stably integrated PARP1 variants. Data are presented as box plots for (*A*) and the median for (*B–D*) from biological triplicate experiments. ∗∗*p* < 0.01; NS, not significant. Scale bars: 10 μm.

In our previous work, MiDAC is reported to occupy DSB-flanking chromatin and is directly involved in DDR, in which DNTTIP1 is the key player in MiDAC engagement (8). However, how MiDAC is recruited to DSBs remains elusive. To elucidate the molecular mechanism that MiDAC localizes to damaged chromatin, we performed laser micro-irradiation assays in the presence of inhibitors of enzymes involved in the DDR. As Figure 1*A* shows recruitment of GFP-DNTTIP1 was not significantly affected by inhibitors of key DNA damage kinases targeting DNA-PKcs NU7026, ataxia telangiectasia mutated (ATM) KU55933, and ataxia telangiectasia and Rad3-related (ATR) VE-821, but was markedly enhanced by PARP1 inhibitors rucaparib and talazoparib, suggesting that DNTTIP1 is possibly co-trapped with PARP1 at DNA lesions.

To further explore the relevance between MiDAC complex and PARP1 at DNA lesions, we use the PALM MicroBeam laser microdissection workstation to induce focal damage, which was labelled with γH2AX. Consistently, PARP1 knockdown severely impaired the accumulation of both DNTTIP1 (Fig. 1*B*) and MIDEAS at damage sites (Fig. 1*C*). This defect was rescued by re-expressing either wild-type PARP1 (PARP1/Wt) or a PARylation-deficient mutant (PARP1/E988K) (18, 22) (Fig. 1, *B* and *D*). PARP1 depletion did not alter the levels of MiDAC proteins, including DNTTIP1, MIDEAS, HDAC1 and HDAC2 (Fig. 1*E*). Collectively, these data support the argument that MiDAC is oriented to DSB-proximal chromatin in a PARP1-dependent but PARylation-independent manner.

### The dimerization domain of DNTTIP1 mediates interaction with PARP1

**Figure 2:**
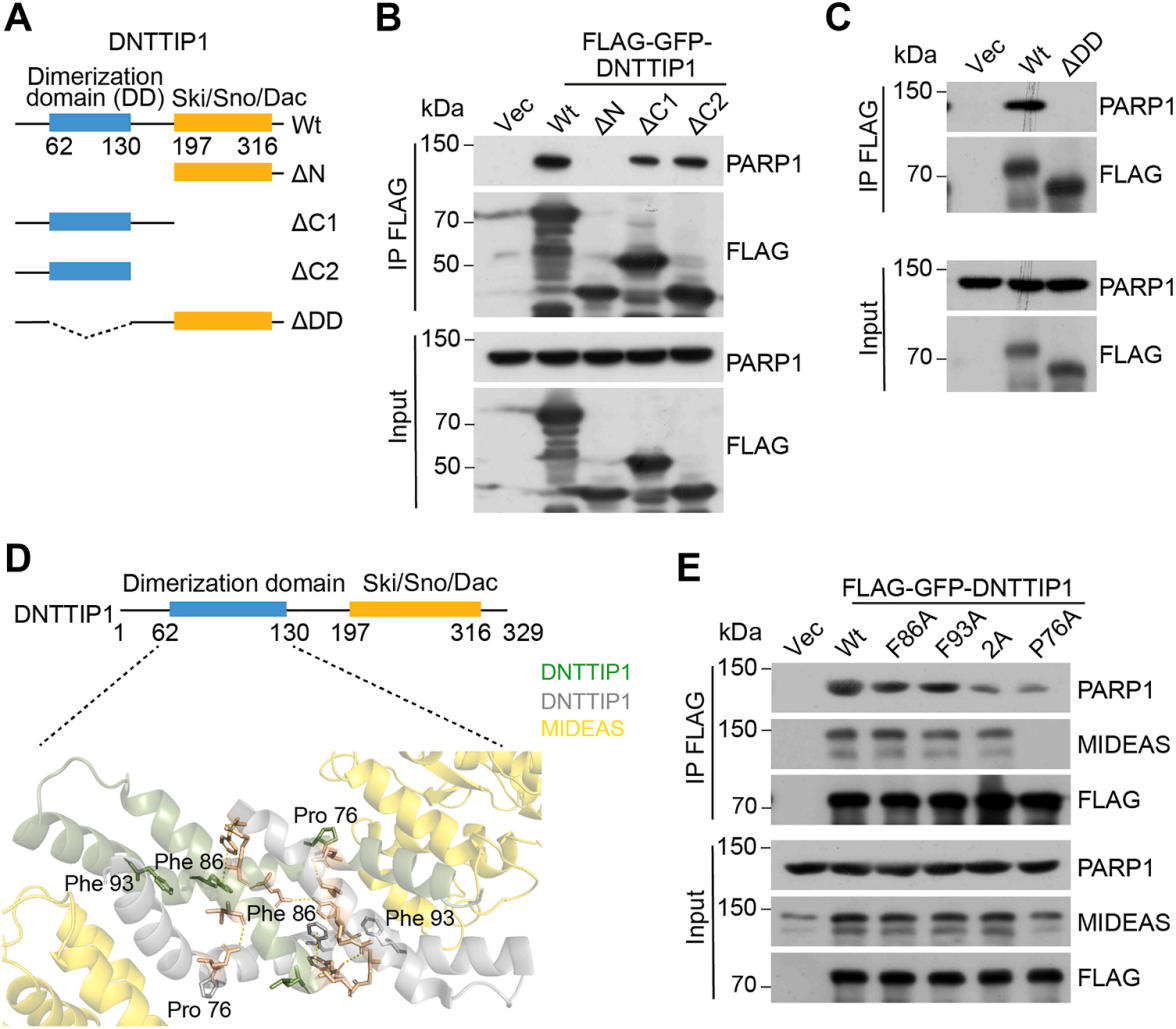
**The dimerization domain of DNTTIP1 mediates interaction with PARP1.***A*, schematic diagrams of the DNTTIP1 variants are shown. *B*, Co-immunoprecipitation (Co-IP) analysis of the interaction between FLAG-GFP-tagged DNTTIP1 truncated mutants and PARP1. *C*, Co-IP analysis of the DNTTIP1 interaction with PARP1 following deletion of the dimerization domain. *D*, the dimerized DNTTIP1-MIDEAS structure (PDB: 6Z2J). MIDEAS is shown in *yellow* and dimerized DNTTIP1 is shown in *green* and *grey*. Potential interactions between dimerization domains are shown in *yellowdashed lines*. The side chains of P76, F86 and F93 are shown in the structure. *E*, Co-IP analysis of the interaction between FLAG-GFP-tagged DNTTIP1 variants, PARP1 and MIDEAS.

DNTTIP1 was identified as a scaffold protein responsible for recruiting the MiDAC complex to damaged chromatin in previous studies (8). Therefore, we hypothesized that PARP1 might recruit the MiDAC complex to damaged chromatin through its interaction with DNTTIP1. DNTTIP1 contains a dimerization domain (DD) and a Ski/Sno/Dac domain. We constructed four DNTTIP1 truncated mutations: deletion of the N-terminal (ΔN), deletion of the C-terminal (ΔC1), a further deletion of the C-terminal beyond ΔC1 (ΔC2) and deletion of the DD (ΔDD) (Fig. 2*A*). Subsequently, we performed a Co-immunoprecipitation (Co-IP) experiment to study the interaction domain between PARP1 and DNTTIP1. Co-IP assays with truncated DNTTIP1 mutants revealed that the DD is necessary and sufficient for binding PARP1 (Figs. 2*B* and S1*A*). Deletion of the DD abolished the interaction (Figs. 2*C* and S1*B*).

P76, together with F86 and F93, in the DD of DNTTIP1 induces kinks in the helix and plays important roles in modulating the backbone trajectory of the dimerized α−helix (5, 7) (Fig. 2*D*). These three residues are also critical for maintaining the higher-order structure of the DD. Given that DNTTIP1 interacts with PARP1 *via* its DD, we constructed the F86, F93, and P76 mutants of DNTTIP1. Co-IP assays revealed that alanine substitution of F86 and F93 in DNTTIP1 (DNTTIP1/2A) specifically disrupted PARP1 binding, while mutation of a structurally important proline (P76A) also indirectly abrogated the interaction, likely by altering complex conformation (Figs. 2*E* and S1*C*).

### MiDAC recruitment and function at DSBs require DNTTIP1–PARP1 interaction

**Figure 3:**
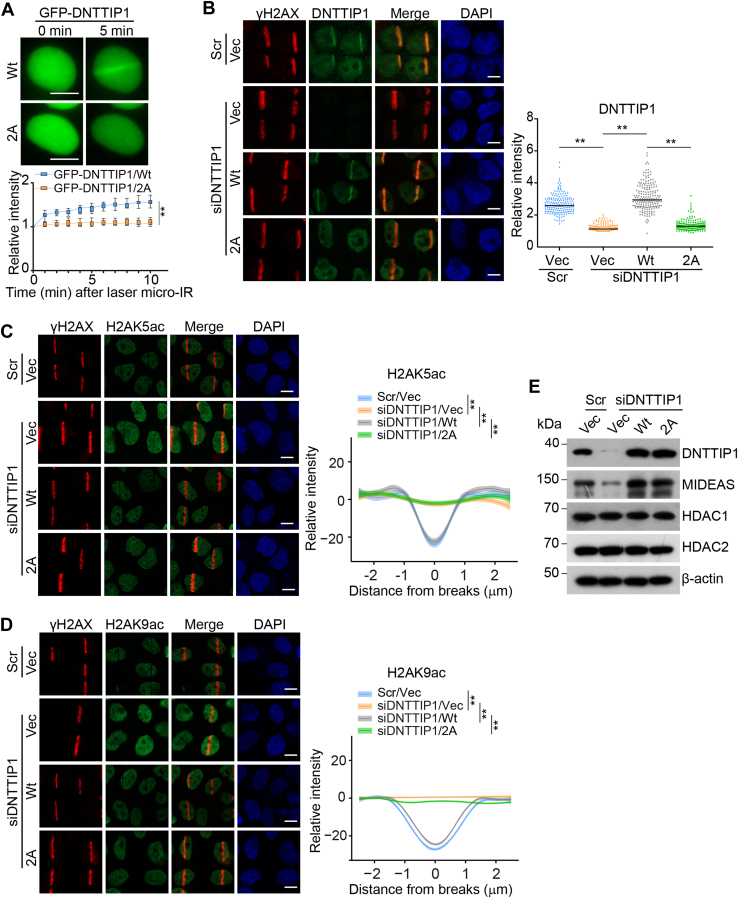
**MiDAC recruitment and function at DSB require DNTTIP1-PARP1 interaction.***A*, recruitment kinetics of GFP-DNTTIP1, as examined by laser micro-IR, in HeLa cells expressing the indicated DNTTIP1 variants. The intensity of the DNTTIP1 signal at the laser stripes was quantified and normalized to the nuclear background intensity (n > 15). Representative micrographs and a quantitative analysis of DNTTIP1 recruitment (*B*), as well as the levels of H2AK5ac (*C*) and H2AK9ac (*D*) around γH2AX-marked laser micro-IR regions, are shown in cells expressing DNTTIP1 3′UTR siRNA and stably integrated DNTTIP1 variants (n > 200). *E*, Western blot analysis of DNTTIP1, MIDEAS, HDAC1 and HDAC2 in cells expressing DNTTIP1 3′UTR siRNA and the stably integrated DNTTIP1 variants. Data are presented as box plots for (*A*), the median for (*B*, *right*), and means ± 95% confidence intervals for (*C*, *right*) and (*D*, *right*) from biological triplicate experiments. ∗∗*p* < 0.01. Scale bars: 10 μm.

Next, to investigate whether the recruitment of MiDAC to DSB-proximal chromatin depends on the interaction between DNTTIP1 and PARP1, laser micro-irradiation was performed. DNTTIP1/2A was found to be impaired in its recruitment to DSBs compared with DNTTIP1/Wt, indicating that DNTTIP1 requires interaction with PARP1 to be recruited to DSB sites (Fig. 3, *A* and *B*).

Bao *et al*. reported that MiDAC targets H2AK5ac and H2AK9ac at DSB-proximal chromatin, where these histone marks respond rapidly to DNA damage and are deacetylated by MiDAC. To investigate whether the removal of H2AK5ac and H2AK9ac following DNA damage also depends on the DNTTIP1–PARP1 interaction, laser micro-irradiation was employed, followed by immunostaining. Depletion of the MiDAC subunit DNTTIP1 efficiently restored the levels of both H2AK5ac (Fig. 3*C*) and H2AK9ac (Fig. 3*D*) along the laser path. Consistent with our previous finding that MiDAC specifically suppresses H2AK5/K9 acetylation at damaged chromatin, this restoration around DNA damage sites could be reversed by re-expressing DNTTIP1/WT; however, DNTTIP1/2A failed to remove H2AK5ac and H2AK9ac from damaged chromatin (Fig. 3, *C–D*). Together, these results suggest that MiDAC acts as a key regulator of H2AK5/K9 deacetylation at DSB-proximal chromatin, a function that depends on the interaction between DNTTIP1 and PARP1.

MIDEAS acts as a central scaffold that maintains the integrity of MiDAC by connecting HDAC1/2 to DNTTIP1. This supports the function of MiDAC at DSB-proximal chromatin and its H2AK5/K9 deacetylation activity. In light of the reported co-dependent expression of MIDEAS and DNTTIP1, we investigated whether disrupting the DNTTIP1–PARP1 interaction impacts MIDEAS expression. As with DNTTIP1/WT, the DNTTIP1/2A mutation restored endogenous MIDEAS levels in DNTTIP1-deficient cells (Fig. 3*E*). This suggests that MiDAC’s deacetylation function at DSBs depends on the DNTTIP1–PARP1 interaction. While MiDAC integrity relies on the co-dependent expression of MIDEAS and DNTTIP1, this co-dependence was not affected by the DNTTIP1/2A mutation.

### Disruption of the DNTTIP1-PARP1 interaction leads to genome instability

**Figure 4:**
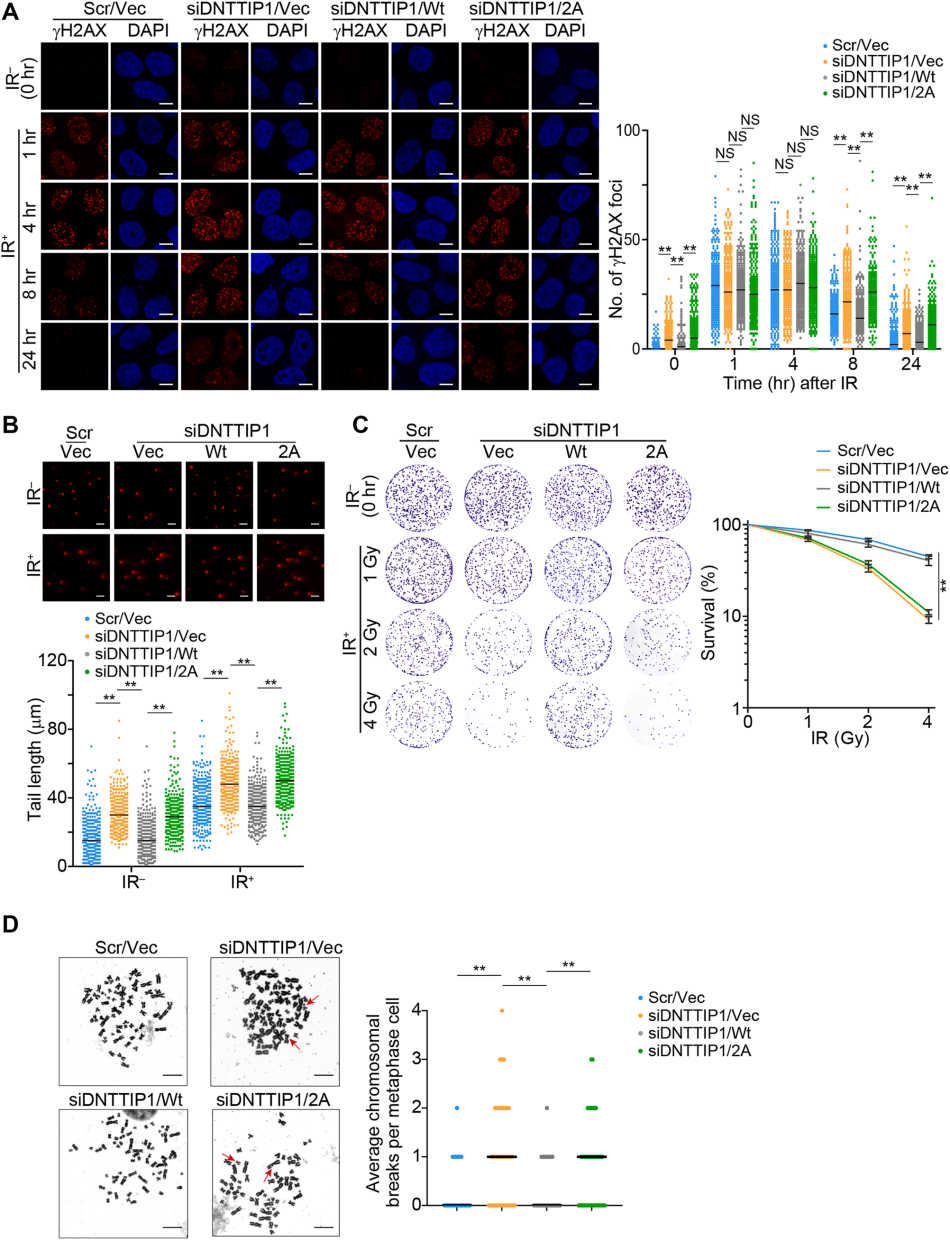
**Disruption of the DNTTIP1-PARP1 interaction leads to genome instability.***A*, γH2AX foci formation was observed by immunostaining and confocal microscopy in HeLa cells expressing 3′UTR siRNA against DNTTIP1 and stably integrating DNTTIP1 variants following treatment with 6 Gy of ionising radiation (IR). The cells were collected at the indicated time points, after which the number of γH2AX foci in each cell was quantified (n > 250). *B*, accumulation of damaged DNA was assessed using a neutral comet assay in HeLa cells expressing 3′UTR siRNA against DNTTIP1 and stably integrated DNTTIP1 variants under control or at 8 h post-6 Gy IR treatment conditions (n > 300). The length of comet tails was quantified and presented. *C*, colony formation of HeLa cells expressing 3′UTR siRNA against DNTTIP1 and the stably integrated DNTTIP1 variants after receiving different doses of IR treatment. The number of colonies in each treatment was quantified and shown. *D*, representative micrographs and quantitative analysis of chromosome/chromatid breaks (*red arrows*) in HeLa cells expressing 3′UTR siRNA against DNTTIP1 and stably integrated DNTTIP1 variants. (n > 50). Data are presented as the median for (*A*, *B* and *D*) and mean ± SD for (*C*) from biological triplicate experiments. ∗∗*p* < 0.01; NS, not significant. Scale bars: 10 μm for (*A*) and (*D*), and 100 μm for (*B*).

MiDAC plays a pivotal role in counteracting DNA damage and maintaining genome integrity. We therefore investigated whether its function is regulated by PARP1. To evaluate the impact of the DNTTIP1-PARP1 interaction on genome stability, we initially examined levels of γH2AX, a chromatin marker of genome instability, and discovered that it increased upon DNTTIP1 knockdown. Consistent with this finding, DNTTIP1-depleted cells displayed increased accumulation of DNA damage and lower efficiency of DNA damage repair after ionizing radiation (IR). These phenotypes were significantly alleviated by the overexpression of wild-type DNTTIP1 (DNTTIP1/Wt), but not by a mutant form (DNTTIP1/2A) (Fig. 4*A*).

Furthermore, neutral comet assays confirmed the accumulation of DNA damage following DNTTIP1 loss. Again, these defects were alleviated by DNTTIP1/Wt overexpression, but not by DNTTIP1/2A (Fig. 4*B*). Overall, these results suggest that the DNTTIP1-PARP1 interaction plays a crucial role in maintaining genome stability and protecting cells from DNA damage. Further colony formation assays revealed that cells expressing the DNTTIP1/2A mutant were significantly more vulnerable to IR compared to those reconstituted with the wild-type protein, underscoring the importance of the DNTTIP1-PARP1 interaction in cellular survival following genotoxic stress (Fig. 4*C*). Karyotype analysis provided further chromosomal-level evidence that depletion of DNTTIP1 caused a significant increase in chromosomal and chromatid breaks. This genomic instability was effectively suppressed by the overexpression of DNTTIP1/Wt; however, the DNTTIP1/2A mutant was unable to do so (Fig. 4*D*).

In summary, these results collectively demonstrate that the ability of the MiDAC complex to counteract DNA damage and preserve genome integrity depends on the physical and functional interaction between DNTTIP1 and PARP1. Disruption to this interaction impairs the capacity for DNA repair, increases chromosomal aberrations and leads to genome instability.

### The DNTTIP1–PARP1 interaction is essential for efficient NHEJ

**Figure 5:**
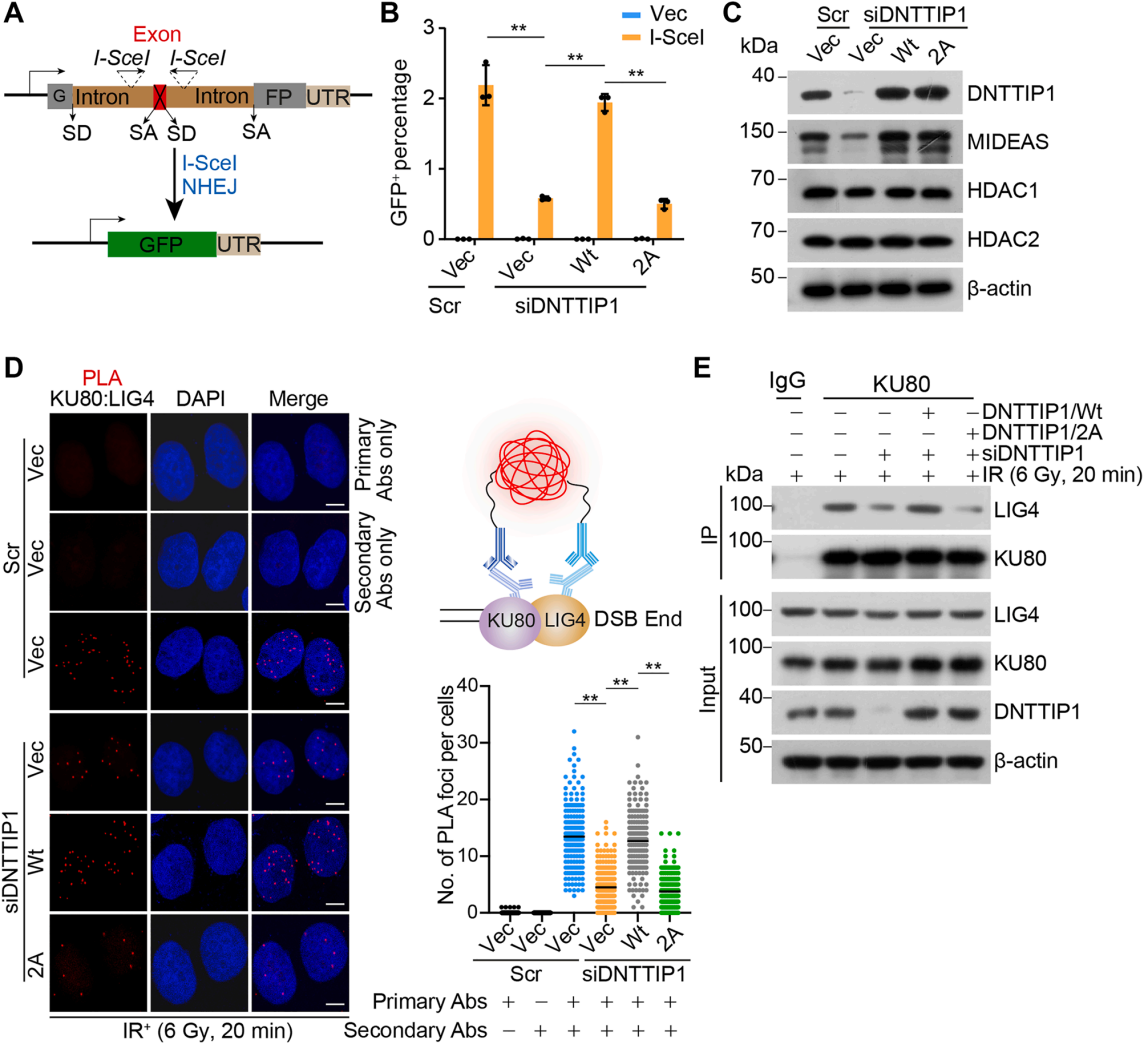
**The DNTTIP1-PARP1 interaction is essential for efficient NHEJ.***A*, NHEJ efficiency, as reflected by the fraction of GFP-positive cells in I-SceI-transfected and DNTTIP1-deficient HeLa reporter cells. A schematic of the splicing-based NHEJ reporter system is shown. *B*, NHEJ efficiency, as reflected by the recombinant GFP fraction, in reporter cells with 3′UTR siRNA against DNTTIP1 and stably integrated DNTTIP1 variants. *C*, MiDAC expression in HeLa NHEJ reporter cells expressing 3′UTR siRNA against DNTTIP1 and stably integrated DNTTIP1 variants. The knockdown effect was examined by IB. *D*, evaluation of the *in situ* interaction between KU80 and LIG4 by PLA assays in cells expressing DNTTIP1 3′UTR siRNA and stably integrated DNTTIP1 variants following IR treatment (n > 250). The specificity of background staining was verified using only the primary antibody or the secondary antibody. Abs, Antibodies. *E*, Co-IP analysis of the KU80-LIG4 interaction in cells expressing DNTTIP1 3′UTR siRNA and the stably integrated DNTTIP1 variants after a 20-min 6 Gy IR treatment. Data are mean ± SD for (*B*) and the median for (*D*); ∗∗*p* < 0.01. Scale bars: 10 μm.

MiDAC promotes genome stability through NHEJ repair. We therefore investigated the role of the DNTTIP1-PARP1 interaction in this pathway. Using a well-established NHEJ reporter assay (Fig. 5*A*), we found that DNTTIP1 knockdown significantly impaired NHEJ efficiency, measured by the proportion of GFP-positive cells (23, 24). This defect was fully rescued by the re-expression of wild-type DNTTIP1 (DNTTIP1/Wt), but not by the interaction-deficient mutant DNTTIP1/2A (Fig. 5*B*). Consistent with its role in stabilizing the MiDAC complex, the reintroduction of DNTTIP1/Wt as well as DNTTIP1/2A-restored endogenous MIDEAS protein levels in DNTTIP1-deficient cells (Fig. 5*C*). Taken together, these results demonstrate that the DNTTIP1-PARP1 interaction is critical for MiDAC-dependent NHEJ repair.

Appropriate DNA end synapsis, regulated by core NHEJ components (KU70-KU80, LIG4, XRCC4, and XLF), is essential for repairing chromatinized DSBs. Since we previously showed that MiDAC facilitates DSB repair by guiding the assembly of the NHEJ synaptic complex on damaged chromatin, we asked whether this function depends specifically on the DNTTIP1-PARP1 interaction. To address this, we used a proximity ligation assay (PLA) (25) to monitor the *in situ* association of KU80 with LIG4 at repair sites, a key step in synaptic complex formation. Consistent with the model that MiDAC modulates this interaction, DNTTIP1 depletion attenuated KU80-LIG4 focus formation. This defect was significantly rescued by overexpression of wild-type DNTTIP1 (DNTTIP1/Wt) but not by the PARP1-binding mutant DNTTIP1/2A (Fig. 5*D*). Co-IP assays on cellular extracts from these cells corroborated these findings (Fig. 5*E*). Therefore, the DNTTIP1-PARP1 interaction is specifically required for MiDAC to guide the assembly of the NHEJ synaptic complex on damaged chromatin and promote DSB repair.

### MiDAC promotes immunoglobulin class switch recombination *via* the DNTTIP1-PARP1 axis

**Figure 6:**
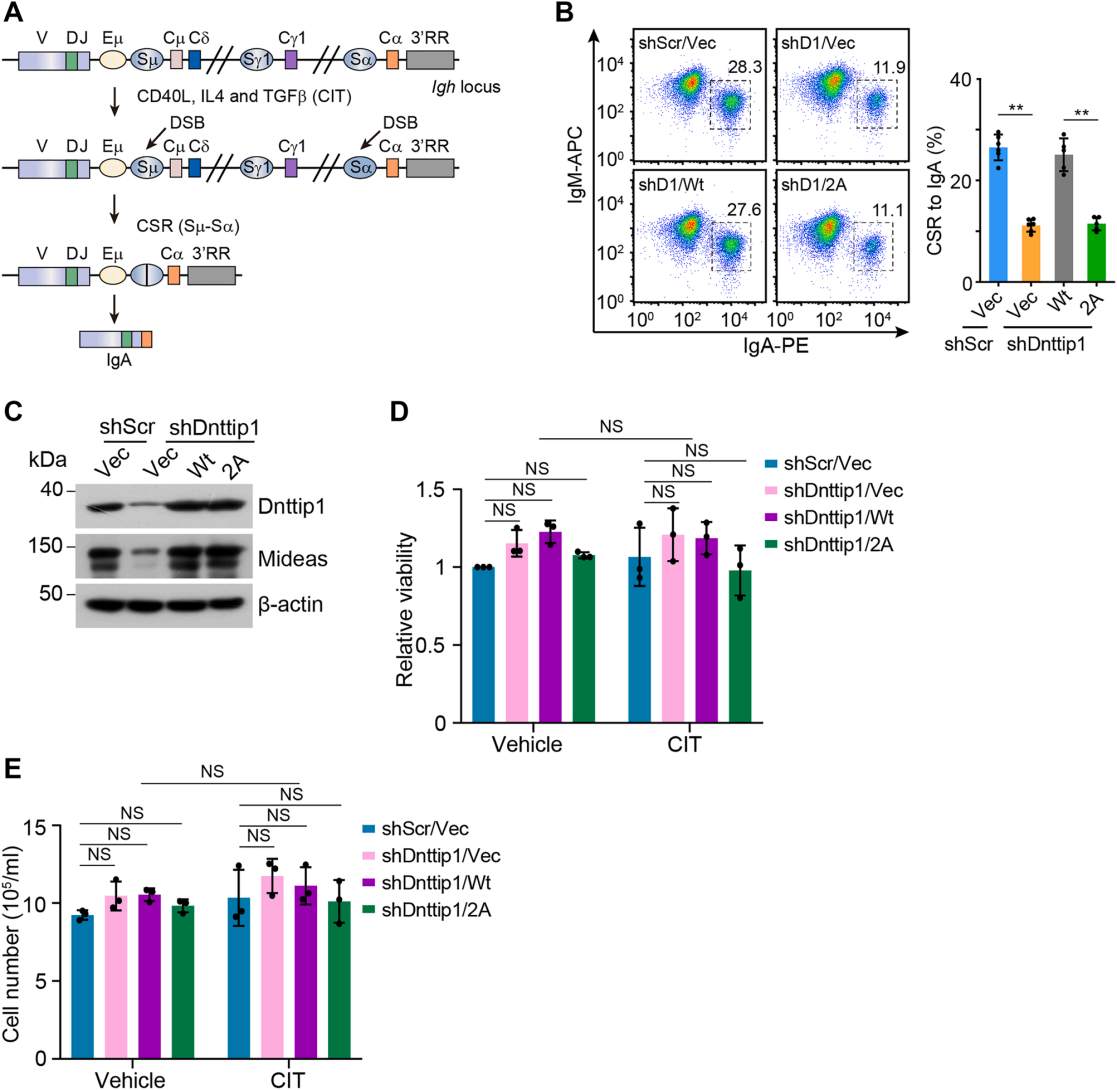
**MiDAC promotes immunoglobulin class switch recombination *via* the DNTTIP1-PARP1 axis.***A*, a schematic showing the IgM to IgA switch. *B*, the efficiency of the IgM to IgA switch in CH12F3 cells with a stable Dnttip1 knockdown that were integrated with either Dnttip1/Wt or Dnttip1/2A (n = 6). *C*, Western blot analysis of Dnttip1 and Mideas expression in CH12F3 cells with Dnttip1 knockdown that were stably integrated with Dnttip1/Wt or Dnttip1/2A. *D*, viability of CH12F3 cells expressing genetically modified Dnttip1 in the absence or presence of CIT stimulation for 72 h. CIT represents anti-CD40, IL4 and TGF-β. *E*, proliferation of CH12F3 cells expressing genetically modified Dnttip1 in the absence or presence of CIT stimulation for 72 h. Data are mean ± SD for (*B*), (*D*) and (*E*) from biological triplicate experiments. ∗∗*p* < 0.01; NS, not significant.

As NHEJ is the primary pathway for immunoglobulin class switch recombination (CSR)-a process involving cytidine deaminase (AID) (26)-dependent DNA breaks at switch (S) regions-we investigated whether the involvement of MiDAC in CSR depends on the DNTTIP1-PARP1 interaction. Using mouse CH12F3 cells, we quantified IgA-switching efficiency after generating lentiviral-based Dnttip1 knockdown cells stimulated with a cytokine cocktail (CD40L, IL-4, and TGF β; CIT) to induce IgA switching (27, 28). Dnttip1 knockdown substantially reduced the IgA-switched population (Fig. 6, *A* and *B*). Critically, this defect was fully rescued by complementation with wild-type Dnttip1 (Dnttip1/Wt), but not by the PARP1-binding mutant (Dnttip1/2A, containing F86A and F93A mutations equivalent to human DNTTIP1/2A) (Fig. 6*B*). Knockdown efficiency was confirmed by immunoblotting (Fig. 6*C*). Importantly, the observed CSR defect was not attributable to general cellular fitness, as the viability (Fig. 6*D*) and proliferation (Fig. 6*E*) of these cells subjected to Dnttip1 knockdown, or those stably expressing either Dnttip1/Wt or Dnttip1/2A, remained unchanged under these conditions (Fig. 6, *D* and *E*). Together, these findings demonstrate that MiDAC is an essential regulator of immunoglobulin CSR. This function is consistent with its established role in promoting the assembly of the NHEJ synaptic complex and critically depends on DNTTIP1-PARP1 binding to ensure MiDAC’s proper localization at damaged chromatin and complex integrity.

Overall, our results show that MiDAC’s role in regulating immunoglobulin CSR is dependent on the DNTTIP1-PARP1 interaction and is independent of the manner of PARylation. Furthermore, this targeted activity is essential for the efficient repair of AID-induced DNA double-strand breaks *via* the NHEJ pathway.

## Discussion

This study reveals a new mechanism for recruiting and activating the MiDAC complex at sites of DNA damage, centered on a direct interaction between PARP1 and DNTTIP1 that is independent of PARylation. We identify PARP1 as the primary sensor that physically anchors MiDAC to chromatin proximal to DNA breaks. This expands the functional repertoire of PARP1 beyond its canonical enzymatic role, emphasizing its significance as a scaffolding protein in the DDR (17, 18).

Our findings that the dimerization domain of DNTTIP1 binds to PARP1 and that point mutations (F86A/F93A) disrupt this interaction without affecting the stability of the MiDAC complex provide a precise molecular tool with which to analyze MiDAC’s DNA repair-specific functions. In addition, we noted that the DNTTIP1/2A mutant did not completely abolish the interaction with PARP1, likely attributable to the fact that the DNTTIP1/2A mutation primarily disrupts the helical kink and backbone trajectory within the dimerization domain of DNTTIP1, thereby affecting its higher-order structure. However, F86 and F93 are not necessarily the direct binding residues for PARP1. We speculate that PARP1 may also interact with other regions of the DD, such as the main chain of the α-helix, which the DNTTIP1/2A mutation may not fully disrupt. The subsequent failure of H2AK5/K9 deacetylation at DSBs in DNTTIP1/2A cells highlights the fact that proper chromatin remodeling at lesions depends on this recruitment mechanism. The resulting genome instability phenotypes-increased DNA damage, radiosensitivity and chromosomal aberrations firmly establish the biological significance of this pathway in maintaining genomic integrity.

A key mechanistic insight is that the DNTTIP1-PARP1 interaction licenses the assembly of the NHEJ synaptic complex, as demonstrated by the defective KU80-LIG4 interaction upon its disruption. This positions MiDAC, through PARP1 tethering, as a critical facilitator of the early steps of NHEJ, potentially by modulating the local chromatin architecture to allow efficient docking and engagement of core NHEJ factors (29). The physiological relevance of this axis is confirmed by its essential role in immunoglobulin CSR, an NHEJ-dependent process (27). The inability of the DNTTIP1/2A mutant to rescue CSR defects directly links this specific protein interaction to adaptive immune function.

In conclusion, we uncover the DNTTIP1-PARP1 interaction as a crucial linchpin connecting DNA damage detection to MiDAC-mediated chromatin modulation and efficient NHEJ. This pathway ensures genome stability and supports vital immune functions. Given the clinical use of PARP inhibitors, our discovery of a PARylation-independent function of PARP1 in NHEJ regulation may have implications for understanding therapy responses and resistance mechanisms.

## Experimental procedure

### Cell culture

HeLa and HEK 293T cells were obtained from ATCC and cultured in Dulbecco's Modified Eagle Medium (DMEM) supplemented with 10% fetal bovine serum (FBS), 100 U/ml penicillin, and 100 μg/ml streptomycin, in accordance with the supplier's protocol. CH12F3 cells were maintained in RPMI 1640 medium containing 10% FBS, antibiotics (100 U/ml penicillin and 100 μg/ml streptomycin), 50 μM 2-mercaptoethanol, 1 × MEM non-essential amino acids, 1 mM sodium pyruvate, and 10 mM HEPES. All cell lines were regularly monitored for characteristic morphology and growth patterns, and confirmed to be free of *mycoplasma* contamination.

### Co-IP

Cells were lysed in NETN buffer (50 mM Tris-HCl, pH 8.0, 150 mM NaCl, 0.2% Nonidet P-40, 2 mM EDTA) supplemented with protease inhibitor cocktail (Roche) for 20 min at 4 °C. Lysates were clarified by centrifugation at 14,000*g* for 15 min at 4 °C. For immunoprecipitation (IP), approximately 500 μg of total protein was incubated with 1 to 2 μg of control or target-specific antibody under constant rotation at 4 °C for 12 h. Subsequently, 50 μl of a 50% slurry of protein G magnetic beads (Invitrogen) was added, and incubation continued for an additional 2 h. Beads were collected on a magnetic stand (Invitrogen) and washed five times with NETN buffer at 4 °C. Bound proteins were eluted by boiling the beads in 2 × SDS-PAGE loading buffer for 5 min. Immunoprecipitated complexes were then resolved by SDS-PAGE and analyzed by immunoblotting with the indicated antibodies.

### Lentiviral production

Lentiviral particles were produced by co-transfecting HEK 293T cells with the transfer vector (encoding DNTTIP1/Wt, DNTTIP1/2A, or shDnttip1) and three helper plasmids: pMDLg/pRRE, pRSV-REV, and pVSV-G. Viral supernatant was harvested 48 h post-transfection, clarified through a 0.45 μm filter, and concentrated by ultracentrifugation. Target cells were transduced with the resulting lentivirus, and stable polyclonal populations were subsequently selected using the appropriate antibiotic.

### Laser micro-irradiation

Laser micro-irradiation was performed using two complementary setups. For live imaging of GFP-tagged proteins, a Leica microscope with a 37 °C heating stage and a 365-nm laser diode (Micropoint, Andor Technology) was employed. A linear damage track was generated at 60% laser power under a 60 × objective. For experiments involving non-fluorescent or untagged cells, irradiation was conducted using a Zeiss Observer Z1 inverted microscope equipped with a PALM MicroBeam laser microdissection workstation (355 nm UV-A laser at 40% power, 40 × objective). These cells were cultured in phenol red-free medium on LabTek II chamber slides prior to irradiation.

### RNA interference

siRNA transfections were performed with Lipofectamine RNAiMAX (Invitrogen) according to the manufacturer's instructions, using a final siRNA concentration of 10 nM. The following siRNAs were used: a non-targeting scrambled control siRNA (D-001810-10), siRNA targeting DNTTIP1 and PARP1 (chemically synthesized by Sigma-Aldrich). The sequences of the siRNAs are provided below:

DNTTIP1 siRNA: *CUAGCAUGUGGUUCUAUAU* (3′UTR siRNA).

PARP1 siRNA: *GAGAGAUUCUGUUGCAUAG* (3′UTR siRNA).

### Immunofluorescence

For immunofluorescence, cells grown on glass coverslips were fixed with 4% paraformaldehyde, permeabilized with 0.2% Triton X-100 in PBS, and blocked in 5% donkey serum containing 0.1% Triton X-100. Samples were then incubated with appropriate primary antibodies, followed by species-matched secondary antibodies conjugated to Alexa Fluor 488 or 594 (Invitrogen). Images were acquired on a Zeiss LSM 900 confocal microscope using a 63 × oil immersion objective. To prevent crosstalk between fluorophores, each channel was scanned sequentially in multi-track mode. Both standard and large-area scans were performed.

### Proximity ligation assay (PLA)

Cells grown on glass coverslips were briefly washed with cold PBS and fixed with 4% paraformaldehyde for 5 min on ice. After fixation, cells were washed and blocked with 10% fetal bovine serum (FBS) in PBS for 1 h at 37 °C in a humidified chamber. Following another wash, cells were incubated with the indicated primary antibodies overnight at 4 °C. Proximity ligation assay (PLA) was then performed using the Duolink In Situ Detection Reagents (Sigma-Aldrich, DUO92101) according to the manufacturer's instructions. Finally, coverslips were mounted with Fluoroshield mounting medium containing DAPI (Sigma-Aldrich). Images were acquired on a Zeiss LSM 900 confocal microscope using a 63 × oil immersion objective and quantified using ImageJ.

### Class switch recombination (CSR) assay

The murine CH12F3 B-cell line was cultured and stimulated for 72 h with a cocktail containing 1 μg/ml CD40L, 5 ng/ml IL-4, and 0.5 ng/ml TGF-β (CIT) to induce switching from IgM to IgA. Cells were then labeled with APC-conjugated anti-IgM and PE-conjugated anti-IgA antibodies for analysis. Flow cytometry data were acquired on a BD LSRFortessa instrument and analyzed using FlowJo software (version 10).

### Antibodies and reagents

The information on the antibodies used in the article is as follows: LIG4 (for IB and PLA; A11432), FLAG (for IB; AE-063),β-actin (for IB; AC004) and HA (for IB; AE-105) from Abclonal; DNTTIP1 (for IB and IF; A304-048A-M), IgA-PE (for flow cytometry; 12-4204-83), IgM-APC (for flow cytometry; 17-5790-82) and CD40 (for CSR; 16-0402-81) from Thermo Fisher Scientific; H2AK5ac (for IF; PTM-106) and KU80 (for IB and PLA; PTM-5405) from PTM BIO; γH2AX (for IF; 05-636) from Millipore; HDAC1 (for IB; YT2112) from Immunoway; H2AK9ac (for IF; ab177312) from Abcam; PARP1 (for IB and IF; sc-7150) from Santa Cruz; HDAC2 (for IB; H2663) and FLAG M2 magnetic beads (M8823) from Sigma. The reagents IL4 (I1020), Puromycin (P8833), KU55933 (SML1109), NU7026 (N1537) and VE-821 (SML1415) were purchased from Sigma; TGF-β (P04202) were purchased from R&D Systems, and Rucaparib (S1098), Talazoparib (S7048) were purchased from Selleck.

### Plasmids

The full-length cDNA of DNTTIP1/Wt was chemically synthesized (You Bio) and cloned into pLVX-FLAG, pLVX-NLS-FLAG-GFP or pLVX-HA vectors. The full-length cDNA of PARP1 was chemically synthesized (You Bio) and cloned into pLenti-FLAG vectors. Mutated and truncated variants were subsequently generated using site-directed mutagenesis and PCR-based DNA recombination methods.

### Comet assay

DNA damage was assessed using the CometAssay kit (Trevigen) according to the manufacturer's protocol. Briefly, cells were resuspended in ice-cold PBS at a density of 1 × 10^5^ cells/ml. A 5 μl aliquot of this suspension was mixed with 50 μl of molten low-melting-point agarose and immediately pipetted onto a comet slide to form a microgel. Slides were then immersed in pre-chilled lysis solution and incubated at 4 °C for 60 min. Following lysis, slides were equilibrated in neutral unwinding buffer at room temperature for 60 min. Electrophoresis was performed in a pre-cooled tank filled with neutral electrophoresis buffer at 1 V/cm and 300 mA for 30 min at 4 °C. Subsequently, slides were rinsed twice in deionized water (5 min each), fixed in 70% ethanol for 5 min, and stained with 100 μl propidium iodide in the dark for 20 min. Comet images were acquired using a Zeiss LSM 900 confocal microscope.

### Colony formation assay

For the colony formation assay, control and irradiated cells were cultured in complete medium for 14 days, with regular medium changes. Subsequently, the cells were washed with PBS, fixed with ice-cold methanol for 10 min, and stained with 0.5% (w/v) crystal violet for 20 min. Colonies consisting of 50 or more cells were counted manually.

### Statistics

All quantitative data were derived from at least three independent experiments and are presented as mean ± standard deviation (SD) or as box plots. For comparisons of bar graphs and box plots, statistical significance was assessed using one-way analysis of variance (ANOVA). For scatter plots, the Mann-Whitney U test was applied. All analyses were performed using IBM SPSS Statistics (version 19.0.0). The specific sample size (n) for each experiment is provided in the corresponding figure legend. Differences were considered statistically significant at ∗*p* < 0.05 and ∗∗*p* < 0.01; "NS" denotes not significant.

## Data availability

All data needed to evaluate the conclusions are presented in the paper.

## Supporting information

This article contains supporting information.

## Conflict of interest

The authors declare that they have no conflicts of interest with the contents of this article.
