# Supplementary material for: The DNTTIP1-PARP1 interaction orchestrates MiDAC recruitment and activity for NHEJ-mediated genome stability
Source: J Biol Chem. 2026 Jul 16;302(9):113342. doi: 10.1016/j.jbc.2026.113342 (PMC13486350; doi:10.1016/j.jbc.2026.113342)
Supplement: Supplementary file 1 [file mmc1.pdf]

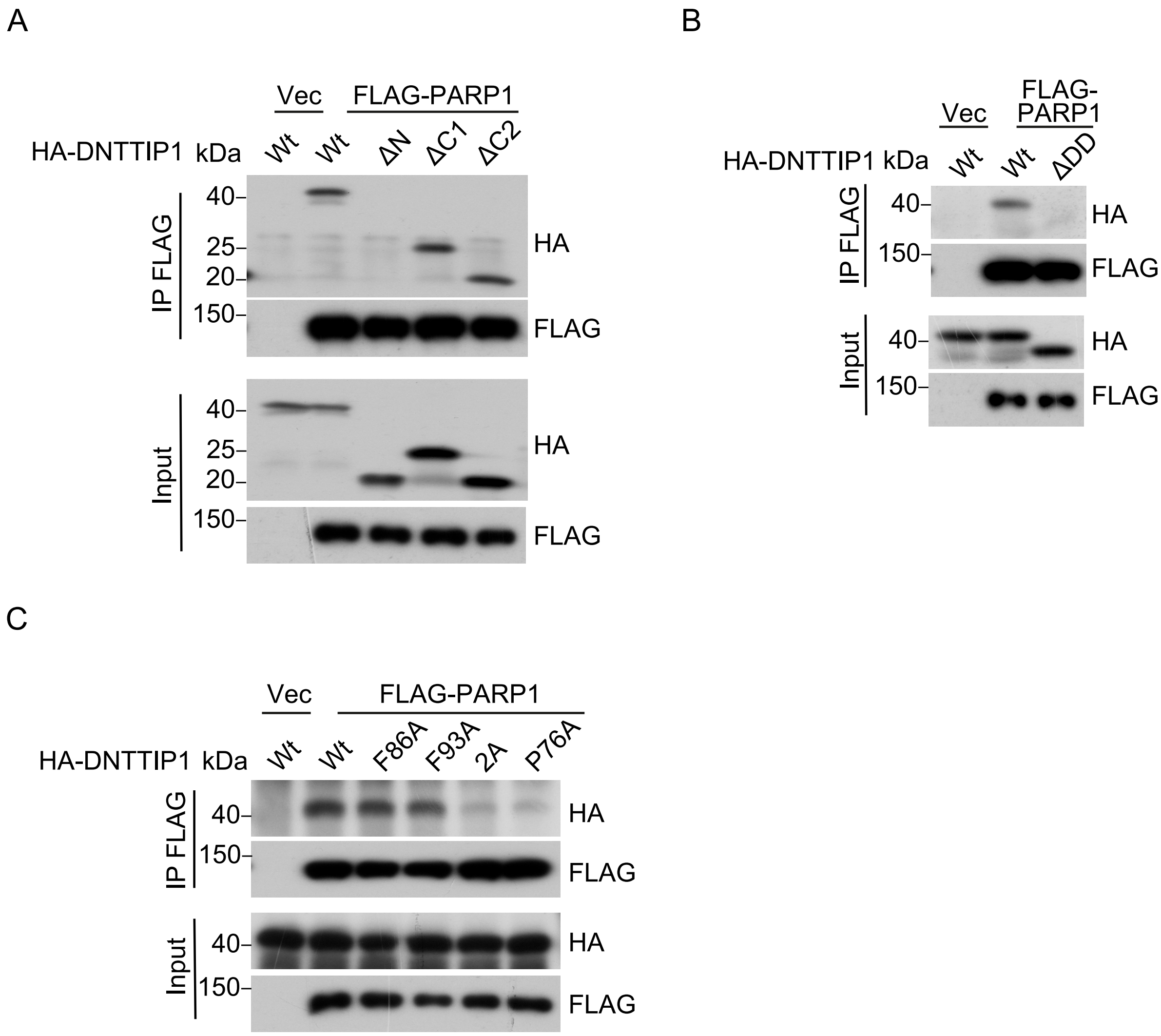

Figure S1. Related to Figure 2. The dimerization domain of DNTTIP1 mediates interaction with PARP1. (A) Co-IP analysis of the interaction between FLAG-PARP1 and HA-tagged DNTTIP1 truncated mutants. (B) Co-IP analysis of the interaction between FLAG-PARP1 and HA-tagged DNTTIP1 with a deletion in the dimerization domain. (C) Co-IP analysis of the interaction between FLAG-tagged PARP1 and HA-tagged DNTTIP1 variants.
